# Analgesic Use After Discharge Following Total Knee Arthroplasty Evaluated Using the Experience Sampling Method

**DOI:** 10.3390/jcm14103506

**Published:** 2025-05-16

**Authors:** Jasmijn E. Willemen, Sanda van Kruining-Kodele, Catherine J. Vossen, Richel Lousberg, Therese A. M. J. van Amelsvoort, Andrea J. R. Balthasar

**Affiliations:** 1 School for Mental Health and Neuroscience, Maastricht University, Universiteitssingel 40, 6229 ER Maastricht, The Netherlands; jasmijn-willemen@live.nl; 2 Section Neuropsychology and Psychopharmacology, Faculty of Psychology and Neuroscience, Universiteit Maastricht, 6229 ER Maastricht, The Netherlands; sandakodele@gmail.com; 3 Neural Injury Group, Nuffield Department of Medicine (Clinical Neurosciences), University of Oxford, Oxford OX1 2JD, UK; 4 Department of Anesthesiology and Pain Medicine, MUMC+, P. Debyelaan 25, 6229 HX Maastricht, The Netherlands; c.vossen@mumc.nl; 5 Department of Psychiatry and Psychology, MUMC+, P. Debyelaan 25, 6229 HX Maastricht, The Netherlands; r.lousberg@maastrichtuniversity.nl (R.L.); t.vanamelsvoort@maastrichtuniversity.nl (T.A.M.J.v.A.)

**Keywords:** experience sampling method, analgesia, postsurgical, acute pain, total knee arthroplasty

## Abstract

**Background/Objectives**: The inadequate management of postsurgical pain represents a major clinical issue, often leading to suboptimal outcomes in the immediate postoperative period and an increased risk of developing chronic postsurgical pain. The present study aimed to examine the relationship between postsurgical pain, mood, and the use of prescribed analgesics after total knee arthroplasty (TKA). **Methods**: This prospective observational explorative study enrolled 28 patients scheduled for TKA between February 2018 and March 2019. Using a digital experience sampling method (ESM) tool that included questions on pain, analgesic use, and both positive and negative effects, patients reported their current status up to ten times daily. The questions were administered over five days following postoperative discharge. Data analysis was performed using descriptive statistics and multilevel regression, accounting for the hierarchical structure of the data. **Results**: On 85.5% of the days post-discharge, the patients did not adhere to the prescribed acetaminophen regimen. Multilevel analyses revealed that the groups who overused or underused acetaminophen reported significantly heightened levels of pain. NSAIDs were generally underused. Post-discharge opioid use decreased over time, with no evidence of abuse. Overall, the non-adherent group reported lower mood levels and higher pain scores than the adherent group. **Conclusions**: Most patients did not adhere to the prescribed analgesics despite experiencing pain. Therefore, clinical interventions should prioritize identifying patient subtypes to tailor analgesic use effectively. This approach will facilitate the development and improvement of personalized acute postsurgical pain treatment protocols, ensuring more precise and effective pain management strategies for patients.

## 1. Introductions

Chronic postsurgical pain (CPSP) significantly affects the quality of life of patients. It is associated with increased healthcare costs, both directly through pain management and indirectly through lost income and out-of-pocket expenses [1]. CPSP exhibits a high comorbidity with psychiatric disorders [2]. In addition, its prevalence ranges from 6% to 30% following total knee arthroplasty (TKA) [3]. This variation in its prevalence can be attributed to factors such as differences in surgical techniques, anesthesia, pain management strategies, and geographical locations [4,5]. Notably, the inadequate control of acute postsurgical pain (APSP) results in unnecessary pain and sleep disturbances. It also increases the risk of developing CPSP, with up to 60% of patients experiencing severe APSP [2,6].

In hospital settings, a numeric rating scale (NRS) is used to report pain scores and adjust the pain treatment if necessary [7]. Pain monitoring typically ceases once patients are discharged. However, the intensity of APSP can increase after discharge in various patient populations postoperatively [8,9]. As early discharge from the hospital is becoming increasingly common for orthopedic patients, it is crucial to understand how these patients manage pain with analgesics at home post-discharge. In the absence of direct patient feedback in a home setting, the effective control of APSP is crucial for supporting recovery.

Inadequate medication use during the acute phase of pain can lead to the development of CPSP for several reasons. First, insufficient pain relief can cause central sensitization of the nervous system, where the nervous system becomes hypersensitive to pain stimuli, increasing the likelihood of chronic pain. Second, neuroplastic changes in the brain can result in the formation of new pain pathways, which further contribute to pain chronification. Finally, unrelieved acute pain can exacerbate emotional distress and alter behavior, causing patients to develop an increased perception of pain and engage in fewer daily activities [10,11]. To prevent pain chronification, a combination of acetaminophen, non-steroidal anti-inflammatory drugs (NSAIDs), and oxycodone is often used to effectively manage postoperative pain [12]. Multimodal analgesia, which combines local anesthetics with different oral analgesics, is currently considered the optimal approach for managing APSP, as it targets various pain pathways [6]. Notably, the inconsistent use of prescribed analgesics is one of the factors contributing to the increased intensity of APSP after discharge [13]. This variability in adherence may be influenced by mood disorders [14]. However, there is limited information available regarding the relationship between the current mood of the patients and their adherence to the analgesic regimen. This knowledge gap could serve as a potential target for intervention during the transition from acute to chronic pain.

Previous pilot studies have evaluated APSP using the digital experience sampling method (ESM) [8,15], revealing the improper use of prescribed analgesics, which adversely affected pain management. The ESM, a real-time diary method, allows for the collection of multiple semi-random daily reports from patients, including information regarding mood, pain levels, and analgesic use throughout the day. This approach provides more detailed insights into the progression of APSP, real-time mood, and behavioral patterns compared to retrospective self-report questionnaires [16]. Based on these studies, we hypothesized that actual analgesic intakes could deviate from the prescribed regimen.

Therefore, the present study aimed to gain insights into the self-management of pain using postsurgical analgesics, including the effects of APSP and mood following TKA, as measured through the ESM. This study aimed to address three main research questions. The first objective was to investigate how patients utilize prescribed analgesics following discharge. The second objective was to explore whether reported pain varies among patient subgroups with different analgesic intake patterns. The third objective was to evaluate the relationship between analgesic adherence, reported pain, and mood. 

## 2. Materials and Methods

This prospective explorative observational study was conducted between February 2018 and March 2019 at the Maastricht University Medical Center (MUMC+) in the Netherlands. The inclusion criteria were as follows: provision of informed consent, scheduled for TKA, aged between 18 and 80 years, fluent in speaking and reading Dutch, discharge around 48 h after surgery, and owning and using a smartphone. The exclusion criteria included unfamiliarity with handling a digital questionnaire, visual impairment, a hospital stay longer than 48 h, and revision of total knee replacement. Orthopedic surgeons approached patients during their outpatient consultations. Upon inclusion, several weeks before surgery, each patient received an informational briefing from an mHealth officer at our hospital, who explained the study and the technical aspects of the app. During the measurement period, mHealth officers were available to support patients with technical issues and address any questions. This study was approved by the Medical Ethical Committee of the Academic Hospital Maastricht and the University of Maastricht (METC), the Netherlands (approval number: 2017-0145; 27 November 2017), and all patients provided written informed consent (Supplement S1).

### 2.1. ESM Tool

The ESM tool is often used in clinical psychology and for monitoring medication adherence in patients with chronic illnesses [17,18]. The digital ESM tool used in this study was the Psymate app (www.psymate.eu), an E-health ESM tool validated by van Os et al. [16]. The app generated ten acoustic alerts daily at semi-random times, prompting the patient to complete a short report on their current state. The use of semi-random timing was crucial for two reasons: to ensure that alerts were evenly distributed throughout the day and to prevent patients from anticipating the next alert, which could lead to changes in their daily routine.

Each alert consisted of 23 questions, which had to be completed within 15 min to be considered valid. The ESM procedure commenced on the day of discharge and lasted for five days. Most patients were discharged home on the second day after surgery and continued their recovery with a physiotherapist visit for standardized physical training. Mood and pain were assessed using a 7-point NRS, with scores as follows: 1 = none, 4 = moderate, and 7 = excessive. The mood was assessed with questions elaborating on positive and negative affects. Table 1 provides a translation of all the ESM questions.

### 2.2. Sample Size

For this exploratory study, we initially planned a year-long data collection period. However, an interim analysis to assess the distribution of potential subgroups and calculate the required sample size was not feasible, as participant enrollment was prematurely halted owing to the COVID-19 pandemic.

Out of the 40 eligible patients, 32 completed the study period successfully without technical problems or delays in discharge within 48 h postoperatively. Patients provided ESM data from postoperative day 3 (the first day after discharge) through day 7, covering five consecutive days after discharge. However, 28 patients were ultimately analyzed as they provided sufficient data (at least 30% of completed short reports). Based on previous research using this ESM approach, a response rate of 30% to the ESM prompts was considered the threshold for inclusion in the analysis [19].

### 2.3. Surgical Procedure for Total Knee Replacement

The orthopedic department of MUMC+ conducts approximately 180 TKAs annually. The patient details are summarized in Table 2. Following anesthetic approval, the patients were electively scheduled for surgery and arrived at the hospital on the day of surgery.

**Figure 1:**
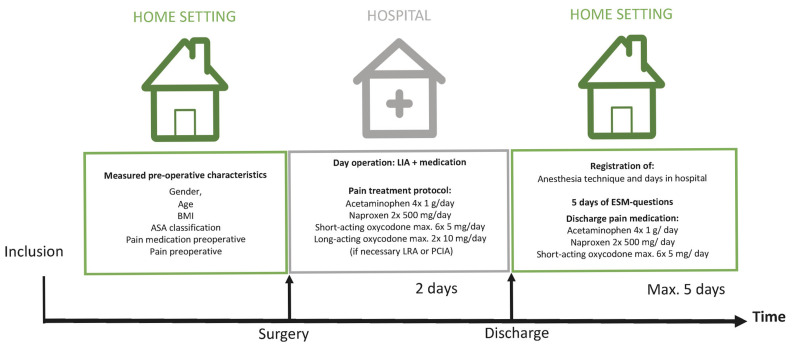
Description of the study course with an indication of preoperative and postoperative registered data. LIA: local infiltration analgesia, LRA: locoregional anesthesia, and PCIA: patient-controlled intravenous analgesia.

The surgery was a standardized procedure, with intraoperative local infiltration analgesia administered to the knee capsule postoperatively [20]. While under care at MUMC+, the participants were managed for APSP using a combination of acetaminophen, NSAIDs (naproxen), and opioids (short- or long-acting oxycodone) (Figure 1).

Patients were discharged based on their mobility, as indicated by the Modified Iowa Level of Assistance Scale score of 0, and the assessment of wound-healing criteria. After discharge, the following analgesics were prescribed: 1 g of acetaminophen, 4 times daily; NSAIDs (500 mg of naproxen), twice daily, depending on comorbidities; and opioids (5 mg of short-acting oxycodone), as needed, up to 6 times daily (Figure 1). The pain experienced during the study period was referred to as APSP.

### 2.4. Statistical Analysis

Statistical analysis comprised two approaches: day-level analysis and short report-level analysis (Supplement S2).

#### 2.4.1. Day-Level Analysis: Descriptive Statistics

Descriptive statistics were employed to analyze the use of acute APSP analgesics after discharge. Initially, the frequency of acetaminophen, naproxen, and oxycodone use per patient per day was evaluated. Deviations from the prescribed analgesic protocol were categorized as inadequate use. Acetaminophen was considered overdosed if more than four 1 g doses were taken per day and underdosed if none or fewer than four 1 g doses were taken. Similarly, naproxen was considered overdosed if more than two 500 mg doses were consumed per day and underdosed if none or fewer than two 500 mg doses were taken. The daily dose of short-acting oxycodone (5 mg) was not classified as adequate or inadequate, as patients were instructed to use it only if acetaminophen and naproxen did not provide sufficient pain relief. However, exceeding the maximum daily dose (more than six 5 mg doses) was considered an overdose. Additionally, the use of acetaminophen and naproxen on days when oxycodone was taken was assessed to ensure adherence to the prescription [6].

#### 2.4.2. Short Report-Level Analysis: Multilevel Statistics

To explore the relationship between acetaminophen intake behavior and pain, a multilevel analysis was conducted at the short-report level for each patient. A new variable was established based on the intake data from the first and last short reports of each day. For each day, acetaminophen intake was examined, and the time between two doses was measured. In this analysis, inadequate acetaminophen use was defined both by the total daily intake (descriptive statistics) and the time interval between two doses.

An adequate intake of acetaminophen was defined as 1 g every 6 h (with a prescribed range of every 4 to 8 h). Dose frequencies exceeding this range were classified as ‘overuse’, while those below this range were classified as ‘underuse’. An underdose was defined as taking a second 1 g dose more than 8 h after the previous dose, and an overdose was defined as taking a second 1 g dose within 4 h of the previous dose. After evaluating acetaminophen intake based on the short reports, patients were classified into underdose, adequate, or overdose groups based on the most frequent intake pattern observed over five days. For example, if an individual took an adequate dose for 2 days but exceeded the prescribed dose for 3 days, they were classified in the “overdose” category.

#### 2.4.3. Multilevel Regression Models

Acetaminophen intake data at the patient level were analyzed using multilevel regression, with short reports nested within participants and within each day. The distribution of ESM pain scores (the dependent variable) was tested for normality using skewness and kurtosis tests. The covariance structure that best fit the data was an autoregressive (AR1) structure. Two models were used to investigate the relationship between analgesic intake patterns and pain. Model 1 was used to compare patients in the adequate acetaminophen group with those in the inadequate groups (underdose and overdose). Model 2 was applied to compare all three groups (underdose, adequate, and overdose) using two dummy variables, with the adequate group as the reference category.

Oxycodone intake was analyzed using multilevel regression, with the pain score as the dependent variable. In Model 3, mood was the dependent variable, with two dummy variables representing the three analgesic groups as predictor variables.

#### 2.4.4. Covariates and Statistical Significance

In all models, the following patient characteristics were used as covariates: age, body mass index, sex, time (short-report number), and time squared. Time squared was included because it best modeled the pain course, indicating that postoperative pain decreases during the day but increases again in the evening. Statistical significance was set at *p* < 0.05. All statistical analyses were performed using SPSS version 28 (IBM, Armonk, NY, USA). Cohen’s d effect sizes were calculated to determine clinical effect sizes.

## 3. Results

The data analysis included a total of 28 patients meeting the inclusion criteria. The general characteristics and statistics of the study population are summarized in Table 2. There were no significant differences in sex (*p* = 0.737) and age (*p* = 0.263) between the study group and the patients excluded from the analysis. Not all patients included in the analysis provided data every day after discharge. Therefore, the total number of short reports per day from days 1 to 5 was 127, 192, 194, 174, and 136, respectively. All 28 patients were prescribed acetaminophen and oxycodone as discharge analgesics. However, owing to medical reasons, only 21 patients were prescribed NSAIDs as discharge analgesics. A total of 823 short reports were collected from all patients during the first 5 days after discharge. As 28 patients were followed up for 5 days, a total of 140 measuring days of analgesic intake were recorded. The mean number of short reports per patient was 29 ± 9.1. The average time taken to complete one short report was 103 s (SD 43.92).

### 3.1. Objective I: Analgesic Use After Discharge

#### 3.1.1. Acetaminophen

Data were collected on the frequency of daily acetaminophen intake among the patients, including instances of overdose, underdose, and adequate usage. Out of the 140 days with available information on acetaminophen intake, acetaminophen was used across all 28 patients on 131 days. Overdose occasions, with 5–9 doses of 1 g of acetaminophen daily, occurred in six patients. On 10 out of the 131 days (7.6%), during which these overdose cases occurred, the average daily dose was 6.7 g. Underdose occasions, with 0–3 doses of 1 g of acetaminophen daily, occurred in 103 out of 131 days (77.9%), across the 28 patients. The average daily underdose was 1.44 g. On 19 out of the 131 days (14.5%), acetaminophen was taken adequately (four doses of 1 g of acetaminophen per day) by 12 patients.

#### 3.1.2. Naproxen

Regarding the frequency of daily naproxen intake, seven patients were not prescribed NSAIDs after discharge. As a result, only 21 patients received a prescription for NSAIDs for 5 days each, resulting in a total of 105 days of NSAID usage. Naproxen was used on 96 out of the 105 days. The results revealed that an underdose of naproxen occurred in 78 out of the 96 days where data about naproxen intake were available (81.3%). This pattern was consistent for all 21 patients to whom naproxen was prescribed. Of the 21 patients who were prescribed naproxen, 10 did not actually take it at all. On 2 out of the 96 days (2.1%), an overdose of naproxen occurred (three to four doses of 500 mg of naproxen per day) in two patients. Naproxen was adequately used by six patients on 16 out of the 96 days (16.7%).

#### 3.1.3. Oxycodone

Patients were instructed to take oxycodone only when acetaminophen and naproxen could not sufficiently control the pain. Therefore, no intake of oxycodone could be classified as an underdose. No overdose of oxycodone occurred, indicating that no patient took more than six oxycodone tablets daily. Furthermore, 9 out of the 28 patients did not take oxycodone after discharge (32.14%). Of the remaining patients who took oxycodone, the average was 1.54 daily intakes. Additionally, oxycodone intake decreased significantly over time (*t* = −3.24, *p* = 0.001).

### 3.2. Objective II: The Relationship Between Analgesic Use and Reported Pain

#### 3.2.1. Acetaminophen

**Figure 2:**
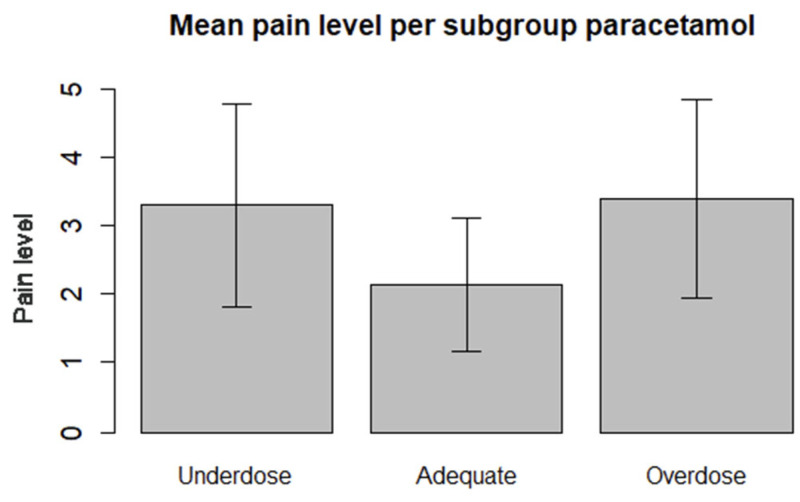
Frequency distribution of the mean pain levels of the different subgroups regarding the use of acetaminophen. Overdose (acetaminophen average of 6.7 g/day), underdose (average acetaminophen of 1.5 g/day), and adequate (3–4 g/day).

There were fourteen, eight, and six patients in the underdose, overdose, and adequate dose groups. Data regarding the mean pain level in each subgroup are presented in Figure 2.

**Figure 3:**
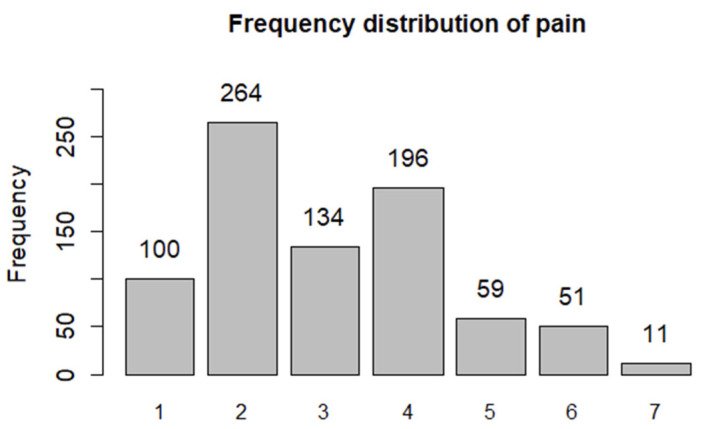
Frequency distribution of the pain scores on a 7-point numeric scale based on the short reports after discharge.

The dependent variable, pain, exhibited an approximately normal distribution (Figure 3). The skewness and kurtosis of the distribution were 0.55 and −0.38, respectively. The mean pain score after discharge was 3.06 ± 1.46. No outliers were observed for the dependent variable. To test whether the mean pain level of the adequate subgroup differed from that of the other two groups, we computed a dummy contrast variable.

Data regarding Models 1 and 2 are presented in Table 3. The first model revealed that adequate acetaminophen intake was significantly negatively related to pain reporting (*t* = −2.96 and *p* = 0.01) compared to the inadequate groups. This result indicated that adequate intake was associated with lower pain scores than those in the other groups.

In Model 2, the inadequate group was divided into two subgroups: patients with an underdose of acetaminophen and those with an overdose of acetaminophen. This analysis revealed that inadequate acetaminophen use was associated with elevated pain intensity compared to the adequate group.

To interpret the effect sizes of the underdose and overdose groups relative to the adequate group, we calculated Cohen’s d. A comparison of the underdose subgroup with the adequate subgroup revealed a Cohen’s d of 0.93, indicating a large effect. Similarly, Cohen’s d for the comparison between the overdose and adequate group was 1.02 [21].

#### 3.2.2. Naproxen

The relationship between naproxen use and pain could not be appropriately analyzed using multilevel analyses, owing to the low frequency of the intake of naproxen.

#### 3.2.3. Oxycodone

We noted an increased pain intensity at the times when patients reported their pain levels after taking oxycodone compared to the times when they did not take it (estimate: 0.15 and *t* = 1.94 and *p* = 0.05).

### 3.3. Objective III: Effect of Mood on Analgesic Use

Both the underdose and overdose groups of acetaminophen exhibited lower mood scores (0.82 and 0.87 points, respectively, on a scale of 1–7) than those of the adequate group. The Cohen’s d effect size fell within the medium-to-large range, indicating a substantial effect. However, no statistical significance was observed (*p* = 0.10 and *p* = 0.24).

We also investigated the relationship between mood and oxycodone use. At the time when patients reported their pain, following the intake of oxycodone since the previous short report, we observed a decrease in the mood scores (0.12 points) on a scale of 1–7 compared to the times when no oxycodone was taken. However, this finding was not significant (*p* = 0.08), and Cohen’s d effect was not clinically relevant.

## 4. Discussion

The present study aimed to gain insights into how patients who underwent TKA adhere to prescribed analgesics after discharge and whether inadequate analgesic use affects pain and mood. Multilevel analyses revealed that the groups who overused or underused acetaminophen reported significantly heightened levels of pain. Overall, the non-adherent group reported lower mood levels and higher pain scores than those of the adherent group.

Considering the overall adherence to APSP analgesic use, we observed a substantial non-adherence percentage, which is consistent with findings from other studies [22,23]. Approximately 80% of patients included in this study exhibited inadequate acetaminophen use. This finding aligns with other studies reporting that acetaminophen was taken as prescribed in less than 50–65% of cases [6,13]. There are various reasons for unintentional and intentional non-adherence, including preferences, fear of addiction, or the perception that they are not experiencing significant pain [24]. However, in the present study, the reasons for non-adherence were not explored, as this was one of the first observational studies to utilize ESM in a postoperative context. Future studies should qualitatively explore patients’ reasons for non-adherence to improve personalized pain management.

Two types of non-adherence are observed: underdose and overdose. In addition to underdosing, the observed acetaminophen overdoses may have been due to various factors. For example, a patient might have underestimated the side effects and toxicity of acetaminophen or may not have remembered when they last used it. However, in the present study, none of the patients required hospitalization due to acetaminophen toxicity. On most days, patients either experienced an underdose of NSAIDs or did not use oxycodone, despite their reported pain. Overall, one-third of the patients did not take any oxycodone at home, and the maximum intake was never exceeded. Consistent with our findings, a previous systematic review concluded that 42–71% of all opioid tablets are unused by postsurgical patients [25]. In contrast, Kalkman et al. reported increases in opioid prescriptions, admissions to addiction clinics, and opioid-related deaths, especially in the Netherlands [26]. The results of the present study suggest that patients prefer to avoid opioid consumption. In contrast to the rising number of opioid prescriptions, the actual intake of opioids following surgery is usually poorly monitored. Therefore, physicians may be unaware if the analgesic is unused, resulting in a potential target for theft, drug diversion, and abuse [25].

Considering the relationship between adherence and pain, our study design cannot establish causality. High pain intensity may contribute to non-adherence; however, the underlying cause of this relationship remains unclear. Increased pain scores were reported both immediately after oxycodone intake and in patients with inadequate acetaminophen intake. However, based on our data, we cannot definitively conclude whether the increased pain scores result from an underdose of medication or if increased pain leads to an overdose of pain medication. Nevertheless, our findings are consistent with another study using ESM in an outpatient orthopedic population [15]. In this previous study, the lowest pain scores were also observed in the group with adequate acetaminophen use, whereas the highest pain scores were observed in the acetaminophen overdose group. These findings contrast with those of Stessel et al., who reported a negative association between APSP and analgesic non-adherence [27]. However, in their study, no ESM was used, and the patients were required to record their analgesic use three times a day.

In addition, we analyzed the relationship between mood and an inadequate intake of acetaminophen or oxycodone. We found that the inadequate group exhibited marginally decreased mood scores and increased pain intensity. Although these results were not significant (*p* = 0.10 and *p* = 0.24, respectively), the observed effect could be considered clinically relevant, as indicated by its Cohen’s d value, which renders the finding somewhat contradictory. This observation is supported by an experimental study by Michaelides and Zis, who concluded that a negative mood increases self-reported pain and decreases tolerance for pain-relevant tasks [28]. In contrast, the opposite effect was observed in patients with a positive mood [29]. Notably, this effect was not observed in patients who took oxycodone.

The novelty of the present study lies in its collection of data about pain and emotional states multiple times a day. Focusing solely on numerical pain measurements overlooks the patient’s acceptable symptom state, highlighting the importance of obtaining additional information, such as affect state and medication use. Our approach allowed for a detailed examination of the relationship between analgesic consumption, pain levels, and mood within a daily context. In contrast, a cross-sectional questionnaire is typically limited in its ability to analyze this dynamic interaction, as it typically involves retrospective data collection at a single time point [30]. Therefore, the use of the ESM in this study enabled a comprehensive view of a patient’s daily behavior and had the distinct advantage of minimizing recall bias. As one of the first studies using ESM postoperatively, a 5-day post-discharge period was selected to balance data richness and patient burden. This duration was deemed feasible, with participants reporting the ESM app as user-friendly and minimally burdensome [31]. Findings support ESM’s feasibility and potential for longer use postoperatively.

Despite the strengths of this study, there were some limitations. First, the total number of patients in this study was relatively low, which may have reduced the reliability of our results. However, a sufficient amount of data was available per patient, increasing the power for within-patient comparisons. While the findings may indicate a possible beneficial effect, the exploratory design of this study limits the strength of any conclusions regarding efficacy. Further confirmatory studies are needed to substantiate these initial observations. Future studies could explore many other aspects and variables that were not investigated in this study, owing to the small study population. For example, these studies can investigate whether the absence of naproxen prescription influenced pain outcomes. Despite the relatively small study population, the main finding remains significant: inadequate postoperative analgesia negatively affects pain, while adequate use is associated with decreased pain and increased mood scores. Second, the reasons for non-adherence were not explicitly assessed in the present study. This limitation could be addressed through a qualitative study that explores the perceptions and underlying beliefs of patients. Third, 4 out of 32 (12.5%) patients did not complete the minimum number of short reports required, which may have introduced a selection bias. Fourth, we used a 7-point NRS instead of the standard 11-point scale that is nearly universally employed in pain research, which may hinder comparisons with the existing literature. However, 7-point scales have consistently been used throughout the history of Psymate. Given the exploratory nature of this study, we chose to maintain this approach.

In future studies, the use of more advanced and technically stable devices might reduce the dropout rates and adjust the pain scale to an 11-pint scale. Finally, the use of analgesics at night was not recorded (outside the short-report window). Therefore, the overdose effect may have been underestimated, while the underdose effect may have been overestimated.

In the present study, pain was used as the dependent variable, acknowledging the bidirectional influence between pain and medication use. Although analgesic usage could have also been selected as a dependent variable, our study specifically focused on the effects of previous medication intake on current pain. Therefore, the inverse relationship could also be explored in a future study. Overall, our results indicate that prescribed analgesics are not frequently taken by patients as directed after discharge, despite experiencing severe pain. This suggests the need for post-discharge follow-up at home when patients are discharged early. Given the lack of patient feedback in the home setting, the effective monitoring and management of APSP is crucial for supporting recovery. A previous study evaluated the effect of postoperative phone-call follow-up versus standard oral and written instructions received after a third molar surgery and indicated an increased adherence score among patients who received phone-call follow-ups [32]. Therefore, phone-call follow-ups could improve analgesic adherence. Moreover, E-health applications, such as an ESM-based application, could be clinically important in the post-discharge setting, enabling timely prompts for patients to contact their healthcare providers or for providers to reach out to patients.

In summary, ESM provides unique insights into the interplay between mood, pain, and analgesic use in patients who have undergone TKA. This approach enables the identification of patient subtypes based on analgesic use, which may be used to develop and improve personalized APSP treatment protocols. Future studies should expand on these findings by investigating interventions aimed at improving postoperative analgesic adherence. These studies could explore tailored interventions, such as personalized pain management plans, based on real-time patient-reported data from ESM applications. Additionally, examining the impact of healthcare provider follow-up strategies, including postoperative phone calls or e-health applications, could further enhance patient engagement and medication adherence during home recovery. These approaches may optimize pain management outcomes and support effective recovery following TKA.

## 5. Conclusions

In conclusion, the present study demonstrated that most patients did not adhere to the prescribed analgesics despite experiencing pain. This study also suggested a relationship between non-adherence and high pain intensity. Notably, the inadequate group of acetaminophen use exhibited decreased mood scores and increased pain intensity. Therefore, clinical interventions should prioritize identifying patient subtypes to tailor analgesic use effectively. This approach will facilitate the development and improvement of personalized APSP treatment protocols, ensuring more precise and effective pain management strategies for individual patients.

## Figures and Tables

**Table 1:** Experience sampling method (ESM) questions, asked up to 10 times a day.

ESM Protocol: Prompt Questions (10 per Day)			
**Domain**	**Item**	**Description**	**Scale**
Mood	1	I feel cheerful	1 = not, 4 = moderate, 7 = very
	2	I feel irritated	1 = not, 4 = moderate, 7 = very
	3	I feel relaxed	1 = not, 4 = moderate, 7 = very
	4	I feel lonely	1 = not, 4 = moderate, 7 = very
	5	I feel anxious	1 = not, 4 = moderate, 7 = very
	6	I feel satisfied	1 = not, 4 = moderate, 7 = very
	7	I feel sad	1 = not, 4 = moderate, 7 = very
	8	I feel guilty	1 = not, 4 = moderate, 7 = very
	9	I feel confident	1 = not, 4 = moderate, 7 = very
	10	I feel stressed	1 = not, 4 = moderate, 7 = very
	11	I feel worried	1 = not, 4 = moderate, 7 = very
	12	Overall, I feel good	1 = not at all, 4 = moderately, 7 = very
Context	13	Physical activity: I	lie down/sit/stand/walk/cycle/exercise/other
	14	What are you doing?	Watching TV/reading/listening to music/using the internet, scrolling through social media/making a phone call/resting/working/doing household chores/maintaining my hygiene/eating, drinking/talking, having a conversation/something else
	15	I do this for	my recovery/my general health/fun/distraction/because I must/for my work/my future/others/other
	16	This is hard for me	1 = not, 4 = moderate, 7 = very
	17	I would rather do something different	1 = not, 4 = moderate, 7 = very
	18	Where are you?	at home/at someone else’s home/at work, at school/healthcare facility/public place/enroute
	19	With whom are you?	no one, alone/partner/family resident/family living away from home/friends/colleagues/acquaintances/strangers
	20	I find this pleasant	1 = not, 4 = moderate, 7 = very
Pain	21	What is your pain level right now?	1 = not, 4 = moderate, 7 = very (at 1 by unpleasant question 23)
	22	Location of the pain	1 = area of operation, 2 = other
	23	Did you take any pain medication since the last short report?	no/heat/cooling/(physio) exercises/paracetamol/ibuprofen or naproxen or diclofenac (voltaren)/tramadol or zaldiar/oxycodone/alcohol/cannabis/other, namely
Physically	24	I am tired	1 = not, 4 = moderate, 7 = very
	25	I suffer from	none of these/drowsiness/nausea/vomiting/itching/dizzy/shortness of breath/palpitations/ringing in the ears/headache/stomachache/muscle pain/difficult bowel movements
	26	This beep bothered me	1 = not, 4 = moderate, 7 = very
			Thank you

**Table 2:** Summary of the general characteristics and descriptive statistics of the patient population.

Patient Characteristics	Frequency/Mean (Standard Deviation)
Sex	11 men and 17 women
Age	61.11 ± 8.23 years
BMI	29.86 ± 5.19 kg/m^2^
ASA classification	I (*n* = 6), II (*n* = 18), III (*n* = 4)
Preoperative pain medication	No (*n* = 14), yes (*n* = 14, including opioids in 4 cases)
Anesthesia technique	17 general, 11 spinal
Preoperative pain score: all patients had >6 months of pain (NRS 1–7)	3.64 ± 1.34
Days in hospital	2 days

ASA, American Society of Anesthesiologists; BMI, body mass index; NRS, numeric rating scale.

**Table 3:** Results of the two models regarding acetaminophen intake across different groups.

Model	Acetaminophen Group	Estimate	Std. Error	*t*-Value	*p*-Value	95% CI (LB)	95% CI (UB)
1	Adequate	−1.62	0.55	−2.96	0.007	−2.76	−0.48
2	Underdose	1.65	0.61	2.71	0.013	0.38	2.91
	Overdose	1.59	0.63	2.51	0.020	0.27	2.90

Adjusted for age, BMI, sex, time, time squared, and side effects. CI, confidence interval.

## Data Availability

Data will be made available upon reasonable request to the corresponding author.

## Supplementary Materials

The following supporting information can be downloaded at: https://www.mdpi.com/article/10.3390/jcm14103506/s1, Supplement S1: Informed consent form; Supplement S2: SPSS syntax.

## Abbreviations

The following abbreviations are used in this manuscript:

APSP	Acute postsurgical pain
CPSP	Chronic postsurgical pain
ESM	Experience sampling method
NRS	Numeric rating scale
NSAID	Non-steroidal anti-inflammatory drug
TKA	Total knee arthroplasty
