# Supplementary material for: Analgesic Use After Discharge Following Total Knee Arthroplasty Evaluated Using the Experience Sampling Method
Source: J Clin Med. 2025 May 16;14(10):3506. doi: 10.3390/jcm14103506 (PMC12112078; doi:10.3390/jcm14103506)
Supplement: Supplementary file 1 [file jcm-14-03506-s001.zip › jcm-3634985-supplementary.pdf]

Maastricht UMC+

Supplement 1: Informed consent

Consent Form

for participation in the scientific study:

Measuring postoperative pain using the PsyMate: Towards improved recovery after surgery.

I have been informed about the study. Furthermore, I have been given the opportunity to ask questions about the study. I have had time to consider my participation. I understand that participation is voluntary and that I have the right to withdraw my consent at any time without having to provide a reason.

I give permission for my (medical) data, as described in the information letter, to be retrieved from my medical records and used for the purposes stated in the information letter. I understand that both my general practitioner and treating physician will be informed about any incidental findings. I give permission for the specialists treating me to be informed that I am participating in this study. I consent to my data being stored for a period of 15 years.

I agree to participate in the study.

Name: ....
Date of birth: ....
Signature: ....
Date: ....

Declaration by the responsible researchers

The undersigned, responsible researchers, declare that the above-mentioned person has been informed both in writing and orally about the aforementioned study. They also confirm that an early termination of participation by the above-mentioned person will have no impact on the care he or she is entitled to receive.

Name: ....
Position: ....
Signature: ....

Name: ....
Position: ....
Signature: ....

Supplement 2 SPSS Syntax

```
* Encoding: UTF-8.
comment Syntax March 2023.
```

Comment based on the reviewers' feedback, we have excluded two patients who were hospitalized for 5 and 6 days (cases: 9008331 and 9008342). Clarify the number of completed beeps per day.

```
FREQUENCIES VARIABLES=dayno_binnen_niet_opgenomen
  /ORDER=ANALYSIS.
```

Comment we have decided to exclude the 14 beeps on day 6 from two individuals and only include data from days 1-5. Additionally, subject 9008330 has only one beep in the home setting, so we have excluded this subject as well.

```
USE ALL.
COMPUTE filter_$=(opgenomen = 0 and subjno <> 9008330 and subjno <> 9008331 and subjno <>
9008342 and dayno_binnen_niet_opgenomen < 6).
FILTER BY filter_$.
EXECUTE.

FREQUENCIES VARIABLES=dayno_binnen_niet_opgenomen
  /ORDER=ANALYSIS.

FREQUENCIES VARIABLES=subjno
  /ORDER=ANALYSIS.

FREQUENCIES VARIABLES= teller_binnen_ppn_niet_opgenomen_sum
  /STATISTICS=STDDEV MEAN
  /ORDER=ANALYSIS.
```

Comment reviewing the general characteristics and descriptive statistics.

```
USE ALL.
COMPUTE filter_$=(opgenomen = 0 and subjno <> 9008330 and subjno <> 9008331 and subjno <>
9008342 and dayno_binnen_niet_opgenomen < 6 and teller_binnen_ppn_niet_opgenomen = 1).
FILTER BY filter_$.
EXECUTE.

FREQUENCIES VARIABLES= geslacht age bmi som_score_surgical_fear som_score_pcs som_pvaq
  ASA pijnmed_preop pain_preop opnameduur
  /STATISTICS=STDDEV MEAN
  /ORDER=ANALYSIS.
```

comment check how many patients provided data each day.

```
USE ALL.
COMPUTE filter_$=(opgenomen = 0 and subjno <> 9008330 and subjno <> 9008331 and subjno <>
9008342 and dayno_binnen_niet_opgenomen < 6).
FILTER BY filter_$.
EXECUTE.
```

```
SORT CASES  BY dayno_binnen_niet_opgenomen.
SPLIT FILE LAYERED BY dayno_binnen_niet_opgenomen.

FREQUENCIES VARIABLES= subjno
  /STATISTICS=STDDEV MEAN
  /ORDER=ANALYSIS.

Comment oxycodone use decreases over time—verify this with GenLink.

USE ALL.
COMPUTE filter_$=(opgenomen = 0 and subjno <> 9008330 and subjno <> 9008331 and subjno <>
9008342 and dayno_binnen_niet_opgenomen < 6).
FILTER BY filter_$.
EXECUTE.

GENLINMIXED
  /DATA_STRUCTURE SUBJECTS=subjno REPEATED_MEASURES=teller_binnen_ppn
COVARIANCE_TYPE=AR1
  /FIELDS TARGET=oxycodon_sinds_vorige_beep TRIALS=NONE OFFSET=NONE
  /TARGET_OPTIONS REFERENCE=0 DISTRIBUTION=BINOMIAL LINK=LOGIT
  /FIXED  EFFECTS=teller_binnen_niet_opgenomen USE_INTERCEPT=TRUE
  /BUILD_OPTIONS TARGET_CATEGORY_ORDER=ASCENDING
INPUTS_CATEGORY_ORDER=ASCENDING MAX_ITERATIONS=100
    CONFIDENCE_LEVEL=95 DF_METHOD=RESIDUAL COVB=MODEL
PCONVERGE=0.000001(ABSOLUTE) SCORING=0
    SINGULAR=0.000000000001
  /EMMEANS_OPTIONS SCALE=ORIGINAL PADJUST=LSD.


Comment OBJECTIVE I

Comment: Now check the frequency distribution of PCM per day.

SPLIT FILE OFF.

USE ALL.
COMPUTE filter_$=(opgenomen = 0 and subjno <> 9008330 and subjno <> 9008331 and subjno <>
9008342 and dayno_binnen_niet_opgenomen < 6).
FILTER BY filter_$.
EXECUTE.


CROSSTABS
  /TABLES=   paracetamol_sinds_vorige_beep BY dayno_binnen_niet_opgenomen BY subjno
  /FORMAT=AVALUE TABLES
  /CELLS=COUNT
  /COUNT ROUND CELL.

Comment check the subject classification for PCM frequency.
```

```
USE ALL.
COMPUTE filter_$=(opgenomen = 0 and subjno <> 9008330 and subjno <> 9008331 and subjno <>
9008342 and dayno_binnen_niet_opgenomen < 6).
FILTER BY filter_$.
EXECUTE.

FREQUENCIES VARIABLES= pcm_foute_inname_thuis
  /STATISTICS=STDDEV MEAN
  /ORDER=ANALYSIS.

Comment check the average pain per PCM group.

MEANS TABLES=pain BY pcm_foute_inname_thuis
  /CELLS=MEAN COUNT STDDEV.


Comment check the distribution of the dependent variable (pain) along with skewness and kurtosis.

USE ALL.
COMPUTE filter_$=(opgenomen = 0 and subjno <> 9008330 and subjno <> 9008331 and subjno <>
9008342 and dayno_binnen_niet_opgenomen < 6).
FILTER BY filter_$.
EXECUTE.

FREQUENCIES VARIABLES=pain
  /STATISTICS=STDDEV MEAN SKEWNESS SESKEW KURTOSIS SEKURT
  /ORDER=ANALYSIS.

comment OBJECTIVE II.

comment comparing the subjective pain between the three PCM groups.

USE ALL.
COMPUTE filter_$=(opgenomen = 0 and subjno <> 9008330 and subjno <> 9008331 and subjno <>
9008341 and and dayno_binnen_niet_opgenomen < 6 and pcm_foute_inname_thuis = -1).
FILTER BY filter_$.
EXECUTE.

FREQUENCIES VARIABLES=pain
  /STATISTICS=STDDEV MEAN
  /ORDER=ANALYSIS.

USE ALL.
COMPUTE filter_$=(opgenomen = 0 and subjno <> 9008330 and subjno <> 9008331 and subjno <>
9008342 and dayno_binnen_niet_opgenomen < 6 and pcm_foute_inname_thuis = 0).
FILTER BY filter_$.
EXECUTE.


FREQUENCIES VARIABLES=pain
  /STATISTICS=STDDEV MEAN
```

```
  /ORDER=ANALYSIS.

USE ALL.
COMPUTE filter_$=(opgenomen = 0 and subjno <> 9008330 and subjno <> 9008331 and subjno <>
9008342 and and dayno_binnen_niet_opgenomen < 6 and pcm_foute_inname_thuis = 1).
FILTER BY filter_$.
EXECUTE.


FREQUENCIES VARIABLES=pain
  /STATISTICS=STDDEV MEAN
  /ORDER=ANALYSIS.

comment model 1 en 2.

USE ALL.
COMPUTE filter_$=(opgenomen = 0 and subjno <> 9008330 and subjno <> 9008331 and subjno <>
9008341 and and dayno_binnen_niet_opgenomen < 6).
FILTER BY filter_$.
EXECUTE.

MIXED pain WITH  adequate_inname_pcm_thuis age bmi geslacht
     teller_binnen_niet_opgenomen teller_binnen_niet_opgenomen_squared  bw_geen_sum
  /CRITERIA=DFMETHOD(SATTERTHWAITE) CIN(95) MXITER(100) MXSTEP(10) SCORING(1)
    SINGULAR(0.000000000001) HCONVERGE(0, ABSOLUTE) LCONVERGE(0, ABSOLUTE)
PCONVERGE(0.000001, ABSOLUTE)
  /FIXED=  adequate_inname_pcm_thuis age bmi geslacht
     teller_binnen_niet_opgenomen teller_binnen_niet_opgenomen_squared  bw_geen_sum
  | SSTYPE(3)
  /METHOD=REML
  /PRINT=SOLUTION TESTCOV
  /RANDOM=INTERCEPT | SUBJECT(subjno) COVTYPE(UN)
  /REPEATED=teller_binnen_ppn | SUBJECT(subjno) COVTYPE(AR1).


MIXED pain WITH  groepfoutdum1_thuis groepfoutdum2_thuis age bmi geslacht
     teller_binnen_niet_opgenomen teller_binnen_niet_opgenomen_squared  bw_geen_sum
  /CRITERIA=DFMETHOD(SATTERTHWAITE) CIN(95) MXITER(100) MXSTEP(10) SCORING(1)
    SINGULAR(0.000000000001) HCONVERGE(0, ABSOLUTE) LCONVERGE(0, ABSOLUTE)
PCONVERGE(0.000001, ABSOLUTE)
  /FIXED=  groepfoutdum1_thuis groepfoutdum2_thuis age bmi geslacht
     teller_binnen_niet_opgenomen teller_binnen_niet_opgenomen_squared  bw_geen_sum
  | SSTYPE(3)
  /METHOD=REML
  /PRINT=SOLUTION TESTCOV
  /RANDOM=INTERCEPT | SUBJECT(subjno) COVTYPE(UN)
  /REPEATED=teller_binnen_ppn | SUBJECT(subjno) COVTYPE(AR1).

comment relation oxycodone and pain.

USE ALL.
```

```
COMPUTE filter_$=(opgenomen = 0 and subjno <> 9008330 and subjno <> 9008331 and subjno <>
9008342 and dayno_binnen_niet_opgenomen < 6).
FILTER BY filter_$.
EXECUTE.

MIXED pain WITH oxycodon_sinds_vorige_beep age bmi geslacht
    teller_binnen_niet_opgenomen teller_binnen_niet_opgenomen_squared  bw_geen_sum
  /CRITERIA=DFMETHOD(SATTERTHWAITE) CIN(95) MXITER(100) MXSTEP(10) SCORING(1)
    SINGULAR(0.000000000001) HCONVERGE(0, ABSOLUTE) LCONVERGE(0, ABSOLUTE)
PCONVERGE(0.000001, ABSOLUTE)
  /FIXED=  oxycodon_sinds_vorige_beep age bmi geslacht
    teller_binnen_niet_opgenomen teller_binnen_niet_opgenomen_squared  bw_geen_sum
  | SSTYPE(3)
  /METHOD=REML
  /PRINT=SOLUTION TESTCOV
  /RANDOM=  INTERCEPT| SUBJECT(subjno) COVTYPE(UN)
  /REPEATED=teller_binnen_ppn  | SUBJECT(subjno) COVTYPE(AR1).

comment relation mood and pcm.

USE ALL.
COMPUTE filter_$=(opgenomen = 0 and subjno <> 9008330 and subjno <> 9008331 and subjno <>
9008342 and dayno_binnen_niet_opgenomen < 6).
FILTER BY filter_$.
EXECUTE.

MIXED mood_global WITH   groepfoutdum1_thuis groepfoutdum2_thuis age bmi geslacht
    teller_binnen_niet_opgenomen teller_binnen_niet_opgenomen_squared  bw_geen_sum
  /CRITERIA=DFMETHOD(SATTERTHWAITE) CIN(95) MXITER(100) MXSTEP(10) SCORING(1)
    SINGULAR(0.000000000001) HCONVERGE(0, ABSOLUTE) LCONVERGE(0, ABSOLUTE)
PCONVERGE(0.000001, ABSOLUTE)
  /FIXED=   groepfoutdum1_thuis groepfoutdum2_thuis age bmi geslacht
    teller_binnen_niet_opgenomen teller_binnen_niet_opgenomen_squared bw_geen_sum
  | SSTYPE(3)
  /METHOD=REML
  /PRINT=SOLUTION TESTCOV
  /RANDOM=  groepfoutdum1_thuis groepfoutdum2_thuis| SUBJECT(subjno) COVTYPE(UN)
  /REPEATED=teller_binnen_ppn  | SUBJECT(subjno) COVTYPE(AR1).

comment relation mood and oxycodone.

USE ALL.
COMPUTE filter_$=(opgenomen = 0 and subjno <> 9008330 and subjno <> 9008331 and subjno <>
9008342 and dayno_binnen_niet_opgenomen < 6).
FILTER BY filter_$.
EXECUTE.

MIXED mood_global WITH oxycodon_sinds_vorige_beep age bmi geslacht
    teller_binnen_niet_opgenomen teller_binnen_niet_opgenomen_squared  bw_geen_sum
  /CRITERIA=DFMETHOD(SATTERTHWAITE) CIN(95) MXITER(100) MXSTEP(10) SCORING(1)
```

```
  SINGULAR(0.000000000001) HCONVERGE(0, ABSOLUTE) LCONVERGE(0, ABSOLUTE)
PCONVERGE(0.000001, ABSOLUTE)
 /FIXED=  oxycodon_sinds_vorige_beep age bmi geslacht
    teller_binnen_niet_opgenomen teller_binnen_niet_opgenomen_squared  bw_geen_sum
 | SSTYPE(3)
 /METHOD=REML
 /PRINT=SOLUTION TESTCOV
 /RANDOM=  INTERCEPT | SUBJECT(subjno) COVTYPE(UN)
 /REPEATED=teller_binnen_ppn | SUBJECT(subjno) COVTYPE(AR1).

MIXED mood_global_lag1 WITH oxycodon_sinds_vorige_beep age bmi geslacht
    teller_binnen_niet_opgenomen teller_binnen_niet_opgenomen_squared  bw_geen_sum
 /CRITERIA=DFMETHOD(SATTERTHWAITE) CIN(95) MXITER(100) MXSTEP(10) SCORING(1)
  SINGULAR(0.000000000001) HCONVERGE(0, ABSOLUTE) LCONVERGE(0, ABSOLUTE)
PCONVERGE(0.000001, ABSOLUTE)
 /FIXED=  oxycodon_sinds_vorige_beep age bmi geslacht
    teller_binnen_niet_opgenomen teller_binnen_niet_opgenomen_squared  bw_geen_sum
 | SSTYPE(3)
 /METHOD=REML
 /PRINT=SOLUTION TESTCOV
 /RANDOM=  INTERCEPT | SUBJECT(subjno) COVTYPE(UN)
 /REPEATED=teller_binnen_ppn | SUBJECT(subjno) COVTYPE(AR1).
```
